# Titanium particles in peri-implantitis: distribution, pathogenesis and prospects

**DOI:** 10.1038/s41368-023-00256-x

**Published:** 2023-11-23

**Authors:** Long Chen, Zian Tong, Hongke Luo, Yuan Qu, Xinhua Gu, Misi Si

**Affiliations:** 1 Stomatology Hospital, School of Stomatology, Zhejiang University School of Medicine, Zhejiang Provincial Clinical Research Center for Oral Diseases, Key Laboratory of Oral Biomedical Research of Zhejiang Province, Cancer Center of Zhejiang University, Engineering Research Center of Oral Biomaterials and Devices of Zhejiang Province, Hangzhou, China; 2 Department of Stomatology, The First Affiliated Hospital, Zhejiang University School of Medicine, Hangzhou, China; 3 Zhejiang University-University of Edinburgh Institute, International Campus, Zhejiang University, Haining, China

**Keywords:** Mechanisms of disease, Metals

## Abstract

Peri-implantitis is one of the most important biological complications in the field of oral implantology. Identifying the causative factors of peri-implant inflammation and osteolysis is crucial for the disease’s prevention and treatment. The underlying risk factors and detailed pathogenesis of peri-implantitis remain to be elucidated. Titanium-based implants as the most widely used implant inevitably release titanium particles into the surrounding tissue. Notably, the concentration of titanium particles increases significantly at peri-implantitis sites, suggesting titanium particles as a potential risk factor for the condition. Previous studies have indicated that titanium particles can induce peripheral osteolysis and foster the development of aseptic osteoarthritis in orthopedic joint replacement. However, it remains unconfirmed whether this phenomenon also triggers inflammation and bone resorption in peri-implant tissues. This review summarizes the distribution of titanium particles around the implant, the potential roles in peri-implantitis and the prevalent prevention strategies, which expects to provide new directions for the study of the pathogenesis and treatment of peri-implantitis.

## Introduction

Peri-implantitis is a prevalent biological complication in the field of oral implantology, and it is challenging to treat. It is mainly manifested by peri-implant soft tissue inflammation and progressive bone resorption.^1,2^ Epidemiological studies have shown that the incidence of peri-implantitis is as high as 20%-47%.^3^ Without appropriate treatment, peri-implantitis can lead to poor treatment outcomes and may even require the removal of the implant. Peri-implantitis has seriously affected patient treatment experience and the broader application of implant therapy. There are numerous risk factors that induce peri-implantitis progression. Due to the driving factors, progression process and clinical manifestations of peri-implantitis are similar to periodontitis, current study considers peri-implantitis to be a class of inflammatory diseases, with bacteria as the initiating factor.^4^ However, compared with periodontitis, peri-implantitis exhibits a larger area of inflammatory cell infiltration and more rapid and severe bone loss.^5^ Bacterial factors alone cannot fully explain the pathological process of peri-implantitis.

**Fig. 1:**
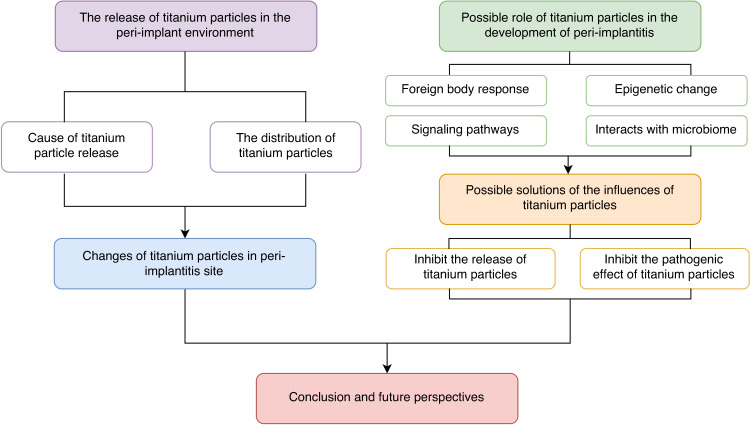
The outline of this review. First, we clarified the causes of the release of titanium particles around the implant and the distribution patterns of titanium particles. Then, we analyzed the changes of the distribution of titanium particles at peri-implantitis site, and summarized the possible mechanism of titanium particles in promoting the development of inflammation and existing prevention and treatment strategies. Finally, we conclude and project future research prospects in this field

Titanium has a long history as an implant material. In the United States, animal experiments on titanium-based implants were conducted as early as 1940, and the first titanium-based dental prostheses were in reported in 1977.^6^ Due to their long fatigue life, corrosion resistance, good biocompatibility and low Young’s modulus, titanium and its alloys have been extensively used in the field of oral implants.^7^ At present, titanium implants are the most widely used commercial dental implants. However, factors such as friction between implant and bone surface, wear caused by biomechanical load, and biological friction corrosion effect inevitably lead to the release of titanium particles in the surrounding tissues.^8^ The 2017 World Workshop on the Classification of Periodontal and Peri-Implant Diseases and Conditions mentioned some evidence of a link between titanium particles and peri-implantitis, but its role as a risk indicator of peri-implantitis remains to be further determined.^9,10^ The role of titanium particles in the progression of peri-implantitis may be underestimated. In the following contents, this article reviews the risk factors influencing the release of titanium particles around the implant, the dispersion of these particles, their potential role in peri-implantitis onset, and possible prevention and treatment methods (Fig. 1). In the first section, we examine the factors impacting the release of titanium particles in the implant’s vicinity. We also analyze the changes in titanium particle distribution at peri-implantitis sites, suggesting a possible role in peri-implantitis development. The second section summarizes potential mechanisms through which titanium particles could affect peri-implantitis progression, offering insights for future treatment approaches. The third section evaluates existing treatment strategies. Finally, we summarize and conclude the paper, addressing current limitations and future research directions. This work aims to provide research insights into the role of titanium particles in peri-implantitis and strategies for its prevention and treatment.

## The release of titanium particles in the peri-implant environment

**Fig. 2:**
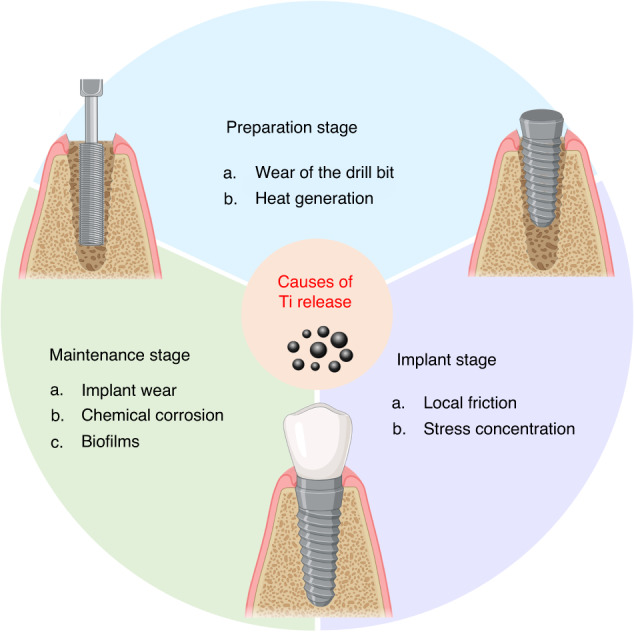
The release of titanium particles in the peri-implant environment. In the preparation stage, the wear of the drill bit and the heat generated during the drilling process release some metal particles. In the stage of implantation, the friction between the implant and bone surface, local stress and other factors lead to further release of titanium particles. In the stage of long-term maintenance of the implant, local wear, micro-movement and micro-gap, chemical corrosion and plaque biofilm interaction contribute to the continuous release of titanium particles

During the process from implantation site preparation to long-term maintenance of implants, titanium particles may overflow. Several studies have detailed this process. For instance, Delgardo-Ruiz et al. ^11^ reviewed the causes of the release of titanium particles and titanium ions during implantation surgery, prosthetic stage and maintenance stage, they identified the risk factors such as mechanical factors, bacteria, saliva, micro-space and fluoride. Romanos et al. ^12^ also observed that titanium particles could be released due to placement, under loading and maintenance factors. Hence, we will further discuss the potential release of titanium particles according to the chronological order of implantation treatment and previous scholarly reviews in order to provide a clear understanding of the cause of the titanium particles release (Fig. 2).

### Cause of titanium particle release around implants

From implant implantation preparation to long-term maintenance, there are many factors that lead to the release of titanium particles around the implant. Friction between drill bit and bone surface and other factors will inevitably lead to the release of titanium particles into the surrounding ground tissues.

#### Implant site preparation stage

Titanium particles are produced during the preparation of the implant site. The most common problems in the process of implant site preparation are wear of the drill bit and heat generation.^13^ During the implant bed preparation, the drill bit is generally used with irrigation (this reduces heat generation), but the wear of the drill bit is unavoidable.

The wear of the implant drill produces metal particles. Rashad et al. ^14^ discovered that the attrition of metal particles occurred during implant site preparation procedure. They compared the effect of several implant system instruments in implant position point preparation. EDS analysis of the water flushed during the operation revealed the presence of Ti, Ag, Cu, Fe, Mn, Zr, Cr and other elements in varying concentrations. Alevizakos et al. ^15^ noticed signs of wear on the main cutting edges of the implant bit after 60 osteotomies on the pig mandible. By drilling into cattle ribs, Allsobrook et al. ^16^ determined that drill corrosion is potentially important in determining the life span of implant burs. Natalia et al. ^17^ found that the depth of drilling was the main factor affecting the temperature change, and no obvious wear was observed after using the drill 50 times. Christian et al. ^18^ investigated the relationship between drill bits, bone density, and temperature increase through in vitro experiments. They discovered that the increased bone density and smaller drill diameter result in a more noticeable temperature rise during surgery, which could significantly affect bone response and drill wear.

Evidence have shown that during the preparation of the implant site, the abrasion of the drill bit against the bone tissue produce metal particles, which may remain around the implant site, therefore replacement used drills may reduce the release of these particles.

#### Implant stage

After the implant was implanted, numerous studies instantaneously detected the presence of titanium particles around the implant. Furranchi et al. ^8^ implanted titanium-based implants in the femur and tibia of sheep to investigate the shedding of titanium fragments from the implant’s surface during initial healing stage. They discovered titanium particles, ranging from 3 to 60 μm in size in peri-implant tissues immediately post-surgery. Barrak et al. ^19^ placed implants in the mandible of pigs and found that metal particles could be detected in the tissue surrounding the implant after implantation immediately. They also found that the larger the diameter of the implant, the more metal particles were released. Additionally, cylindrical implants released more metal particles than conical implants. Guan et al. ^20^ simulated the mechanical changes during implant implantation and discovered areas of stress concentration around the implant. These areas were potential sites for the generation and release into the bone tissue.

The appearance of titanium particles immediately after implant placement, predominantly caused by local friction and stress concentration, seems unavoidable at present. We can only hope that future advancements in implant materials and surgical procedure optimization will alter this phenomenon.

#### Long-term maintenance stage

Titanium particles remain in the tissue surrounding the implant during long-term maintenance after implantation. In the late stage of implant implantation, titanium particles will be released into the surrounding tissues due to chemical corrosion, micro-gap and micromovement, and other reasons

##### Implant wear

Micromotions and fretting damage at the implant/bone interface are overlooked due to limitations in inspection methods, but it is particularly important for the long-term existence of the implant.^21^ The micro-gap and micro-movement between the implant and the upper crown connection, the conventional mechanical cleaning of the implant, and the destruction of the titanium oxide protective layer will promote the increase of implant wear and eventually lead to the gradual release of titanium particles from the implant to the surrounding tissues. The micro-gap and micro-movement between the implant and abutment are caused by the production error of the implant, masticatory load and other reasons, leading directly or indirectly to microleakage and mechanical wear.^12,22^ Patricia A Lopes et al. found small defects ranging from 0.5 to 5.6 μm on different regions of the abutment surface. These defects resulted in the micro-gap in the connection between the implant and the abutment, affecting the adaptability of the implant and the abutment.^23^ Kai Blum et al. applied a force of 98 N to various implants and abutments for 100 000, 200 000, and 1 million cycles, respectively. They found micro-gap between the implants and abutments, with gap size increasing with cyclic loading. All implants showed wear regardless of the interface design.^24^ In addition, micromotion between the implant and abutment will be generated under the action of chewing force, further increasing the micro-gap between the implant and abutment components, which will lead to microleakage, material wear, titanium particle release, and screw loosening.^25–27^ The friction caused by micromotion can also damage the oxide layer on the surface of the titanium-based implant, leading to the release of titanium particles. When the titanium implant is exposed to the air, a layer of oxide film will form on the implant surface. This improves the implant’s corrosion resistance and provides good biocompatibility, promoting wound healing and cell growth at the implant site.^28–30^ However, wear of the implant can destroy the oxide film and exposure the underlying metal, making it more susceptible to corrosion and releasing titanium particles and titanium ions.^31^

Micro-gap and micro-motion are inevitable. Clinicians should aim to choose the implant system with smaller micro-gap, and implant manufacturers should provide relevant data to allow clinicians to make a more informed choice.

In addition, Alrabeah et al. ^32^ analyzed the effects of platform-matched and platform-switched implants on metal particle and ion release levels. They found that titanium had the highest release rate due to corrosion, irrespective of the substrate size or connection mode. The daily maintenance of the implant and prevention and treatment of implant related diseases can also contribute to increased implant wear, eventually leading to the release of titanium particles. Prevention and treatment of peri-implant mucositis and peri-implantitis involve cleaning the implant surface exposed to the oral environment using mechanical and chemical methods. This process generates a significant amount of tiny titanium particles into the surrounding tissue.^33^ During implant decontamination, metal scrapers and ultrasound can effectively remove plaque biofilm, but also cause the release of titanium particles from titanium implants.^34^ Anna Louropoulou et al. evaluated the effects of various instruments on titanium surfaces and found that non-metallic instruments caused less damage to smooth and rough titanium surfaces. Metallic instruments can cause severe damage to the smooth titanium surface, while non-metallic instruments cause less damage.^35^ Valerie Ronay et al. evaluated the cleaning efficiency of commonly used implant debridement methods by simulating non-surgical treatment of peri-implantitis in vitro, SEM results showed that the morphology of titanium surface changed significantly after scaling and ultrasonic treatment.^36^

These findings suggest that clinicians should consider not only the effect of implant decontamination and inflammation resolution in the daily maintenance and prevention of implant-related diseases post-implantation, but also the impact of treatment methods on the implant’s condition. Improper treatment can cause further damage to the implant, release more titanium particles, and potentially lead to peri-implant diseases.

##### Chemical corrosion

Chemical corrosion is also an important cause of titanium particle leakage. Mechanical wear causes damage to the oxide layer of the implant surface, making the implant more susceptible to corrosion. In addition, the complex environment in the oral cavity, including constant changes in temperature, PH, bacteria and other components, and exposure to various chemical agents such as fluoride toothpaste, increase the risk of implant corrosion, resulting in the release of titanium into the environment around the implant in forms such as particles and ions.^37,38^

Sutton E Wheelis et al. discovered that an acidic environment and friction could significantly alter the surface roughness of titanium and promote its corrosion.^39^ Chen et al. found that under fluoride ion conditions, the titanium oxide film barrier was destroyed and the corrosion resistance of pure titanium decreased with increasing fluoride concentration. The sensitivity to corrosion of pure titanium increased after exposure to high fluoride concentration, thereby accelerating the release of titanium ions. This effect was even more pronounced in acidic environments.^40^ Chen et al. studied Ti-6Al-4V dental implants and found that its corrosion and by-products were significant causes of bone loss around implants. This corrosion effect was exacerbated in a fluorine-rich environment.^41^ Barbieri et al. immersed implants with different surface treatments in human saliva and detected that titanium began to be released from all implant surfaces after seven days, with the release increasing over a period of six months.^42^ Leonardo P Faverani et al. studied the effects of carbamide peroxide, hydrogen peroxide, and cola beverage on the surface morphology of commercially pure titanium (CP-Ti) and Ti-6Al-4V, and found that the surface morphology of CP-Ti exposed to 35% hydrogen peroxide changed significantly.^43^ Oral pathogens can adhere to and colonize the titanium surface, leading to its corrosion and degradation.^44^ The titanium particles and titanium ions produced after corrosion of titanium implants can further aggravate the corrosion process. Alhamad et al. found that high concentrations of titanium in saliva can increase the corrosion rate of titanium implants, posing a potential risk of increased implant associated adverse tissue reactions.^45^

Based on current research, there is a mutually reinforcing relationship between the chemical corrosion of implants and the release of titanium particles. Implant corrosion may result in the release of titanium particles. The increased concentration of titanium around the implant can create a more corrosive external environment. Moreover, under inflammatory conditions, the corrosion sensitivity of the implant escalates, an issue that can be mitigated by altering the surface microstructure of the implant.^46^ However, the corrosion of implants, the release of titanium particles and the emergence of inflammation all have mutual reinforcing effects. Determining the initiating factor and the mechanism of the entire process requires further research.

##### Biofilms

Contrary to titanium-based prostheses in large joint arthroplasty, dental implants are exposed to the complex oral microenvironment. Bacteria adhering to the implant surface can easily form biofilms which can influence the implant in various ways.

Sridhar et al. inserted implants in bacterial culture medium and observed that the acidity of the media promoted implant corrosion causing discoloration, roughness, pitting and severe surface rust.^47^ Mathew et al. discovered that lipopolysaccharide (LPS) in bacteria accelerated the exchange between titanium ions and saliva, which diminished the corrosion resistance of titanium and increased its surface roughness.^48^ The bacteria on the implant surface not only trigger an inflammatory immune response, but also initiate electrochemical changes on the titanium surface, specifically corrosion, which aggravates the inflammatory response. Safioti et al. also found higher titanium concentrations in plaque from tissues surrounding peri-implantitis.^49^ The titanium dioxide’s electrostatic force and ionic bond on the implant surface can attract bacterial adsorption. The interaction between the metal surface and the oral environment could lead to the release of implant degradation products into the peri-implant environment, thereby increasing the environmental stress of the microbial community.^1^

Given that bacterial biofilms on the implant surface can cause a series of adverse effects, various methods are employed for implant surface decontamination during daily maintenance. However, these actions could also harm the implant itself and lead to the release of titanium particles. Techniques such as mechanical removal, laser treatment, photodynamic therapy, air polishing, ultrasonic treatment, chemotherapy, electrochemical therapy and the use of antibacterial drugs effectively remove bacterial biofilms. However, these methods could also negatively impact the implant, which could be considered a form of implant wear to a certain extent.^34^

The bacterial biofilm on the implant surface can promote the release of titanium particles and contribute to the development of peri-implantitis. Currently, no uniform standard exists for the removal of bacterial biofilms, a process which could inadvertently damage the implant. Therefore, the optimization of implant decontamination methods and the establishment of uniform standards are essential areas for future research.

Numerous factors can cause the release of titanium particles from the implant, from the preparation stage prior to implantation to the long-term maintenance post-implantation. The wear and tear of implants, chemical corrosion, bacterial biofilm and other factors on the implant surface can all result in surface damage. These factors rarely exist in isolation; instead, they interact, complicating the study of preventative strategies for the release of titanium particles around implants. Furthermore, the titanium particles released from implants have significant effects on peri-implant tissues. They are closely associated with the progression of peri-implantitis and other related diseases. As such, this topic warrants increased attention.

### The distribution of titanium particles

The release of titanium particles is inevitable during implant treatment. Metal particles of different sizes are scattered in the tissue around the implant. Understanding the distribution and location of titanium particles released from implants is crucial for comprehending their role in t implant-related diseases.

Studies indicate that titanium particles are primarily found around implants, epithelial cells, connective tissue, macrophages, and inside bone.^50^ Flatebø et al. ^51^ analyzed the mucosal tissue around the implant six months after implantation, discovering a significant increase in the concentration of titanium particles in the adjacent oral mucosal epithelial tissue. Similarly, He et al. ^52^ investigated the release of titanium particles from human mandibular implants and found that the intensity of titanium increased with distance from the implant, specifically between 556 and 1 587 μm. They also discovered particles ranging from 0.5 to 40 μm in size in human mandibular bone marrow tissue 60 to 700 μm away from the implant. In addition, titanium particles were found in the peri-implant soft tissues, submucosal plaques, and distant lymph nodes.^53^ They can also be transferred systemically through blood supply. Ann Wennerberg et al. implanted implants in rats and traced nanoparticles on the implant surface, and these particles could be detected in blood, liver and other parts.^54^ Research shows that titanium particles can diffuse through the plasma proteins or macrophages in the blood, eventually reaching the lung, spleen, liver or distant lymph nodes.^55^ Conversely, Guglielmotti et al. injected a solution containing TiO_2_ particles into the abdominal cavity of rats, micron-sized titanium particles were detected in the gingiva, suggesting that titanium from other parts of the body can also reach the gingiva through specific pathways.^56^

Titanium particles released from implants can be disseminated throughout the body. However, the potential systemic diseases this phenomenon may cause remain uncertain.. In the tissues surrounding implants, titanium particles can be found in alveolar bone, gingiva, macrophages, etc., which must be considered when studying the progression of implant-related diseases such as peri-implantitis. Despite their presence in the environment around dental implants, the role of titanium particles in the development of peri-implantitis.

## Changes of titanium particles in peri-implantitis site

Existing studies confirm the release of titanium particles into the surrounding tissues from titanium implants, but the impact of these titanium particles on the development of peri-implantitis is still a contentious issue. The following article will analyze the distribution of titanium particles in the peri-implant tissues in patients with peri-implantitis. By identifying the differences, we aim to uncover the potential relationship between titanium particles and the progression of peri-implantitis.

To explore the distribution changes in titanium particles at the site of peri-implantitis, we used ‘peri-implantitis’ and ‘titanium particles’ as search keywords in PubMed database. Our survey of the existing studies (Table 1) revealed that all investigations detected titanium particles at the peri-implantitis site, primarily within the gingival tissue around the implant. Additional findings highlighted the presence of titanium particles in the alveolar bone tissue,^57^ gingival tissue,^58^mucosae tissue^57,59^ and deeper implant site.^60^ Some studies used tissue samples from patients with periodontitis^58,61^ as controls, while others utilized tissue from healthy implants^49,59,60^ for comparison. Prior reviews have shown no significant difference between the distribution of titanium particles in patients with peri-implantitis and healthy individuals. Nevertheless, significant variations were observed in the size and concentration of titanium particles detected across different experiments. It was consistently found that the concentration of titanium particles was significantly higher at the site of peri-implantitis. In contrast, the sources of titanium particles in periodontitis patients were significantly less than those in peri-implant patients due to the absence of titanium-based implants. This discrepancy suggests that titanium particle distribution data obtained from some studies might not reflect the situation accurately at the inflamed sites around the implant. Therefore, these results still warrant further discussion.. While we observed that only a limited number of studies used tissues from around healthy implants as control groups for comparison, many studies merely collected tissues from around inflammatory sites of implants to analyzed the distribution of titanium particles. This may be attributed to the challenges in acquiring tissues from around healthy implants in clinical work and the difficulty in gathering an adequate sample size for comparative study. For future research, it is recommended that some gingival tissue can be collected during the second surgery in the course of implantation treatment. Alternatively, part of the surrounding tissue of the implant can be collected during the surgical removal of implants due to non-inflammatory reasons. This would allow the analysis of the patten of titanium particle release after standard implant procedures, enhancing our understanding of the role of titanium particles in the process of peri-implantitis.

**Table 1:** Relevant study on the correlation of titanium particles at the peri-implantitis sites: Compared with periodontitis patients and the tissues around healthy implants, the concentration of titanium particles at the site of peri-implantitis was significantly higher, and the titanium particles were distributed in the gingival, mucous membrane, bone and other tissues around the implant, with different concentrations and sizes

Study	Number of patients	Detection method	Ti concentration		Ti particle size	Ti particle location
			Peri-implantitis	Control		
Pettersson et al. ^58^	13 peri-implantitis and 11 periodontitis patients	LM、SEM、TEM	(98.7 ± 85.6) μg·g^−1^	(1.2 ± 0.9) μg·g^−1^	(10.9 ± 35.7) μm^2^	Gingival tissue around Ti implant
Mia Rakic et al. ^61^	39 peri-implantitis cases and 35 periodontitis as control	LM、H&E Staining、dispersive X-ray spectrometry	/	/	(8.9 ± 24.8) μm^2^	Free inter-cellular content in peri-implant tissue
Fretwurst et al. ^57^	12 peri-implantitis patients’ biopsies	LM、radiation X-ray fluorescence spectroscopy	3 × 10^5^in bone 7 × 10^5^in soft tissue (area scan)	/	/	Seven bone samples, five mucosal samples
Daubert et al. ^60^	21 peri-implantitis and 24 healthy implants	ICP-MS	(0.2 ± 0.6/0.5) ng sample	(0.1 ± 0.2/0.5) ng sample	/	Deepest site for each included implant
Wilson Jr et al. ^125^	36 peri-implantitis patients’ biopsies	LM、SEM	0.6% titanium element in samples	/	9-54 μm	Peri-implant tissue, Ti was surrounded by inflammatory cell
Pettersson et al. ^126^	3 peri-implantitis patients’ biopsies	ICP-MS	7.3 to 38.9 μmol·L^−1^	/	/	Peri-implant gingiva
Safioti et al. ^49^	Submucosal plaque from 20 implants with peri-implantitis and 20 healthy implants	ICP-MS	0.85 ± 2.47	0.07 ± 0.19	48.73 ng·μL^−1^	Submucosal plaque from implants
Olmedo et al. ^59^	15 peri-implantitis and 15 healthy implants	LM、ICP-MS	2.02 × 10^−9^-2.44 × 10^−9^	0.41 × 10^−9^-0.88 × 10^−9^		Mucosae with and without peri-implantitis
Nelson et al. ^127^	patients who had either titanium implants exhibiting severe peri-implantitis	μ-XRF、 Nano-XRF	/	/	several micrometers to 100 nm	Soft tissue biopsies
Berryman et al. ^128^	10 peri-implantitis patients tissue biopsies	SEM-EDS	titanium wear particles in 90% of the samples	/	/	Peri-implant tissue

Many studies have primarily focused on examining the size, concentration and location of titanium particles around the implant. Results consistently show a significantly higher concentration of titanium particles at the inflammatory site around the implant compared to normal sites. However, t findings vary greatly across different studies concerning particle concentration, distribution location and size. This variability may result from the broad distribution of titanium particles and the differences in the sample location and size across research groups. It may also be related to variation in peri-implantitis severity among individuals. Complicating matters further, the lack of standardized measurement process leads different research teams to adopt varying standards for assessing the distribution of titanium particles at the peri-implantitis site. Some studies measure titanium particle concentrations, while others detect titanium element content. The use of different detection methods and measurement units impedes the comparison outcomes, making it challenging to explore the role of titanium particles in the development of peri-implantitis. Future research should consider establishing unified standard for the detection of titanium particles around the implant, including stipulations on samples collection range and the categorization of collected gingival, mucous membrane and bone tissue etc. In addition, the time of implant function, clinical manifestations and the differences between patients themselves should also be taken into account. Standardized sampling will greatly help to study the mechanism of action of titanium particles in the development of peri-implantitis.

In conclusion, due to the disparities in the research results on titanium particles and the insufficiency of sample size, it is challenging to explore the role of titanium particles in the development of peri-implantitis. It remains difficult to determine how titanium particles contributes to peri-implantitis, understand the governing laws, identify the effective concentration and size, and locate where the distribution of these particles is impactful. These lingering questions necessitate future exploration. In the subsequent section, we will review the extant literature on the role of titanium particles in the progression of peri-implantitis and summarize the potential influences, hoping to offering insights for further study of the role of titanium particles in the progression of peri-implantitis.

## Possible role of titanium particles in the development of peri-implantitis

**Fig. 3:**
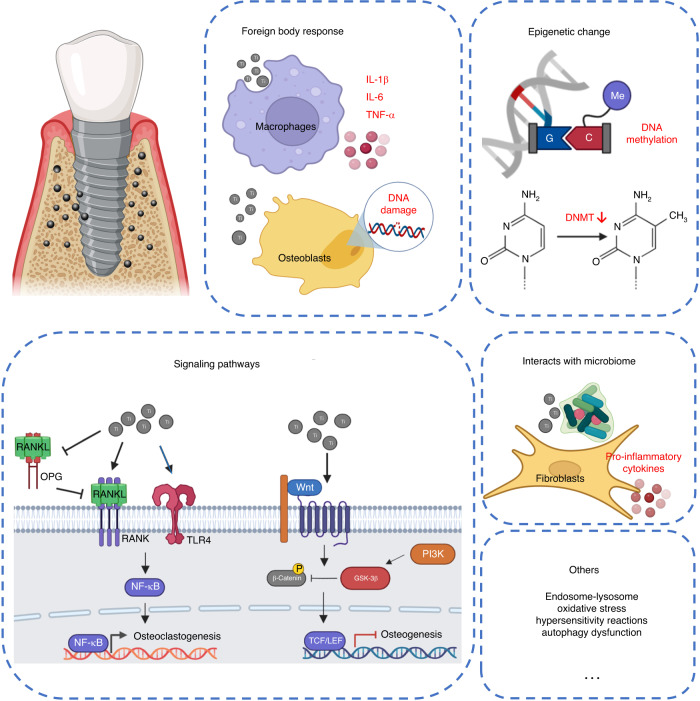
Possible role of titanium particles in the development of inflammation. Foreign body reaction: The presence of titanium particles can induce body foreign body reaction, macrophages phagocytize titanium particles, polarize to M1 type, release pro-inflammatory factors, and induce cell DNA damage; Epigenetics: Titanium and titanium oxides can affect the methylation level of cellular DNA cytosine-5MC by regulating the expression of DNA Class A transferase (DNMT), and subsequently affect the inflammatory response; Signaling pathway: Titanium particles can affect the development of inflammation through a variety of signaling pathways, and regulate NF-κB signaling pathway to affect inflammation by regulating the ratio of RANKL/OPG and affecting the expression of TLR protein family. Wnt/β-catenin signaling pathway is a negative feedback regulatory pathway of titanium-induced inflammation, which can inhibit the NLRP3 inflammasome induced by titanium particles, inhibit inflammation and osteolysis. microbiome: Bacterial corrosion can promote the release of titanium particles, and the combined stimulation of titanium particles and bacteria can promote the expression of inflammation in the tissues around the implant. Others: Oxidative stress, autophagy and other factors may also contribute to the inflammation induced by titanium particles

While the current research on the impact of titanium particles on the development of peri-implantitis remains contentious, a large number of scholars have investigated the potential role of titanium particles. Many studies highlight the inflammatory effect of titanium particles. Furthermore, titanium particles produced by wear and tear after artificial joint replacement can cause chronic inflammation. In the following part, we will summarize the potential ways in which various forms of titanium particles can influence the development of peri-implantitis, and tried to provide new directions for subsequent research (Fig. 3).

### Foreign body response

Foreign body reaction refers to the response of macrophages and allogeneic giant cells to inflammation and wound healing following the implantation of medical devices, prostheses or biomaterials.^62^ Many scholars argue that titanium particles as a foreign particle, like other metal particles, instigate a foreign body reaction in the host, thus promoting inflammation. They do not suggest a specific role for titanium itself.

The submicron and nanoscale fragments released through the degradation of dental implants are perceived as t foreign bodies that trigger the human immune system, thereby inducing an immune response. Studies have shown that the high concentration of titanium particles at the nanoscale can amplify the inflammatory response of surrounding cells.^63^ Macrophages are critical to foreign body reactions as they are precursors to the multinuclear giant cells, a key feature of the condition. The polarization of macrophages affects the entire stage of inflammatory development.^64^ In vitro experiments suggest that titanium ions or particles might have toxic or pro-inflammatory effects, and the introduction of titanium particles can instigate M1 type polarization of macrophages, heighten the expressions of inflammation related genes TNF-α, IL-1β, IL-6, RANKL and induce bone loss during bone integration in mouse models.^65^ Ege et al. ^66^ also confirmed that when titanium particles exist as foreign bodies, they are absorbed by macrophages, which then release pro-inflammatory cytokines, IL-1β and IL-6 and TNF-α. These cytokines promote the development of inflammation, potentially leading to osteolysis. Titanium nanoparticles in living organisms can form biological complexes rich in calcium and phosphate, as well as the crystal structure of some hydroxyapatite. When further internalized by osteoblasts, these particles can induce DNA damage.^67^ However, other studies collecting peri-implantitis samples found no TPS-phagocytic macrophages or multinuclear giant cells, providing no direct evidence of foreign body reaction induced by titanium particles.^61^

Overall, there is much uncertainty surrounding the concept of foreign body reaction. First of all, titanium implants experience normal wear, and the presence of foreign particles can also be detected in tissue samples around many healthy implants. The simplistic view of foreign body reaction cannot adequately explain why these particles do not induce inflammation. Secondly, although titanium and titanium oxides, silver, zinc oxides and other particles can induce inflammation, the effect vary, and some particles can cause DNA damage, which is closely related to the size of the particles. More evidence is needed to support the idea that titanium particles influence the development of peri-implantitis through foreign body reactions.

### Epigenetic change

Epigenetic changes are closely related to the development of inflammatory diseases, and numerous studies have demonstrated that titanium particles can induce epigenetic changes. The main effect of titanium particles on the development of inflammation is DNA methylation.

Daubert et al. ^60^ found a rise in DNA cytosine-5MC methylation level in patients with peri-implantitis, and the methylation level was positively correlated with the concentration of titanium particles, suggesting that DNA methylation may be affected by titanium dissolved products, thereby affecting the development of peri-implantitis. In contrast, Lu et al. ^68^ found that exposing monocytes (THP-1) to TiO_2_ or CuO nanoparticles led to a significant downregulation of DNA methyltransferase (DNMT), reducing DNA methylation expression in a dose-dependent manner. Studies demonstrated that TiO_2_ nanoparticles can induce dose-dependent DNA hypomethylation in peripheral blood monocytes at non-toxic concentrations.^69^ Other studies analyzed the correlation between miRNA changes induced by titanium, silver, zinc and oxides and mRNA expression across all metal types, showing that the genes with the most highly correlated genes were those related to cell cycle regulation, inflammatory response and response to metal ions.^70^ Furthermore, studies have shown that smooth and moderately rough titanium surfaces have distinct impacts on epigenetic changes.^71^ Numerous studies have investigated titanium-induced epigenetic changes, but the conclusions remain inconsistent.^72^

In general, epigenetic changes are likely to be triggered by metal nanoparticles rather than being specific to titanium. Moreover, the difference of titanium particle size and titanium oxide may have different effects on DNA methylation, adding complexity to the study of titanium particle’s potential contribution to the development of peri-implantitis through epigenetic effects. From the previous research, we know that nanometer to micron-level titanium particles and TiO_2_ are present around the implant, necessitating further investigation in this field.

### Signaling pathways

Few studies have delved into changes in signaling pathways involved in the development of peri-implantitis due to titanium particles. Considering the pathogenic role of wear titanium particles after large joint replacement, we have reviewed the signaling pathways that may be influenced by titanium particles, thereby promoting the inflammation development.

#### NF-κB / RANKL/OPG Signaling pathway

Nuclear factor kappa-B (NF-κB) plays a crucial role in cellular responses to external stimuli, which include cytokines, radiation, heavy metals, viruses, etc. This factor is integral to the process of cellular inflammation and immune response. The receptor activator of nuclear factor-kappa Β ligand (RANKL) is a NF-κB activated receptor ligand. Osteoprotegerin (OPG) acts as a decoy receptor homolog of RANKL, inhibiting RANK by binding RANKL, and as such, is intimately involved in the regulation of NF-κB signaling pathway.^73,74^

Titanium and titanium oxides promote osteoclast activation and the development of chronic inflammation by activating NF-κB signaling pathway. These substances may even influence other body parts and contribute towards the onset of allergic bronchitis and cardiac inflammation.^75–78^Takanori Wachi et al. ^79^ found that under LPS stimulation, titanium ions synergistically increased the expression of cytokine CCL2 and RANKL/OPG ratio in gingival tissue. In addition, titanium ion alone elevated the expression of Toll-like receptor 4 (TLR-4) in the gingival epithelium, potentially increasing the sensitivity of gingival epithelium to microorganisms in the oral environment. Studies have analyzed the transcriptomics and proteomics of relevant samples, and confirmed that alterations in biological processes, such as immune/inflammatory or stress responses and TLR signaling pathways are associated with titanium.^72^ TLR4 promotes alveolar bone absorption by regulating RANKL/OPG expression ratio and differential inflammatory cytokine production.

The NF-κB/RANKL/OPG Signal pathway significantly influences the activation of osteoclasts and inflammation development, and is the primary focus in the study of inflammation induced by titanium particles.

#### Wnt/β-catenin Signaling pathway

The Wnt-secreted protein family regulates cell growth, differentiation, function and death, and plays an important role in the process of osseointegration.^80^ Typical β-catenin-dependent signaling leads to activation of T cell factor/lymphocyte-enhancing factor (TCF/LEF). Macrophages use a molecular mechanism for Wnt signaling that modifies their activity, cytokine production and phagocytosis.^81^ The classical Wnt signaling pathway can restrict proinflammatory overactivation via pathogen-associated molecular patterns.^82^ Abaricia et al. ^83^ studied Ti surface-cultured macrophages and found that the mRNA of Wnt ligand was up-regulated in a surface-modified dependent manner. Macrophages serve as an essential source of Wnt ligands during inflammation and healing. Activation of the Wnt/β-catenin signaling pathway can prevent osteolysis induced by titanium particles and shield the damage of osteoblastogenesis.^84,85^ Zichuan et al. ^86^ found that melatonin can regulate the balance between receptor activator of nuclear factor kappa-B ligand and osteoprotegerin through activating Wnt/β-catenin signaling pathway to inhibit the osteolysis induced by titanium particles. The upregulation of Sirtuin 3 can suppress the NLRP3 inflammasome induced by titanium particles through the GSK-3β/β-catenin signaling pathway and promote osteogenesis.^87^ Phosphorylation of GSK-3β lessens the degradation of β-catenin and facilitates the translocation of β-catenin from cytoplasm to nucleus, mitigating the inhibitory effect of titanium particles on osteogenesis.^88^

The Wnt/β-catenin signaling pathway is mainly connected with the osteogenesis process, and can targeted to inhibit the inflammation and osteolysis induced by titanium particles in future studies. In aseptic large joint inflammation, previous studies have attempted to inhibit the development of inflammation induced by wear particles by regulating the Wnt/β-catenin signaling pathway. This method could be a potential treatment for peri-implantitis, pending further research confirmation.

#### Other signaling pathways

The phosphatidylinositol 3-kinase (PI3K)/AKT/ Rapamycin (mTOR) signaling pathway plays a significant role in regulating cell survival, proliferation, growth, metabolism, angiogenesis and metastasis.^89^ In large joint replacement, PI3K/Akt pathway is one of the signal transduction pathways that mediates the activation of macrophages by wear particles. Inhibiting the expression of PI3K can decrease the activity of alkaline phosphatase (ALP), the expression of osteogenic protein Runx2 on titanium surface, thereby affecting osteogenic ability.^90^ Furthermore, inhibition of p110δ, a member of PI3Ks family, can significantly inhibit the expression of TNF-α and IL-6, both associated with inflammation induced by titanium particles.^91^ Xian et al. ^92^ found that titanium particles could promote macrophages autophagy and induce apoptosis through PI3K/Akt signaling pathway. Melatonin can activate butyrate/GPR109A signaling pathway to slow down inflammation and osteolysis. It does this by inducing butyrate enrichment, which activate its receptor GPR109A, inhibiting the activation of NLRP3 inflammasome induced by titanium particles.^93^ Additionally, Crocin induces M2 polarization of macrophages by inhibiting p38 MAPK signaling pathway, thereby inhibiting titanium-induced inflammation.^94^

In conclusion, numerous studies have explored the mechanism of titanium particle-induced inflammation and osteolysis, leading to the development of potential therapeutic drugs targeting these signaling pathways. However, these studies mostly focus on the inflammation induced by titanium wear particles after orthopedic implant surgery. Whether the therapeutic methods identified can be applied to peri-implantitis requires further investigation.

### Interacts with microbiome

As a recognized initiating factor of peri-implantitis, titanium particles may interact with the changed bacterial environment around the implant, promoting the development of peri-implantitis. Studies suggest that titanium corrodes in the presence of bacteria in clinical practice.^95^ Many studies have explored the interaction of titanium particles with bacteria.

Diane et al. ^96^ analyzed the bacterial composition and the distribution of titanium particles deep within peri-implantitis sites. They found that peri-implantitis was associated with a significant increase in Veillonella. Large amounts of dissolved titanium were found at 40% of the sample sites, suggesting a connection between the presence of titanium and peri-implant disease status. Irshad Muhammad et al. ^97^ found that the combined stimulation of titanium wear particles and Pseudomonas gingivalis could promote the expression of genes related to inflammation in peri-implant granulation tissue fibroblasts, thus advancing the development of peri-implantitis. Under bacteria corrosion, the release of titanium particles and ions on the surface of titanium increases,^98^ suggesting a mutual promotion between the two. However, contrasting research shows that nanoparticles can inhibit bacterial activities by disrupting the integrity of bacterial cell membranes, inducing oxidative stress responses, causing protein and DNA damage, and inhibiting DNA replication by binding to DNA.^99^ This conflicting impact makes studying the interaction between bacteria and titanium particles complex.

Some studies have evaluated the characteristics of bacterial growth on the titanium implant surface and its corrosion effect on the implant, but little is known about the infiltration and accumulation of metal particles in the biofilm surrounding the implant and their possible forms of action.^100^ All of this indicates the interaction between bacteria and titanium particles still requires significant exploration.

### Others

Several studies suggest that titanium particles may influence the development of peri-implantitis from lesser-explored perspectives. Martin et al. ^101^ investigated the specific gene expression of inflammatory diseases around oral implants and found that titanium particles may promote the inflammation by activating endosome-lysosome and oxidative stress pathways. Furthermore, the release of titanium particles by implants could potentially enhance antibiotic resistance by altering the microenvironment around the implants. Fernando Suarez-Lopez Del Amo et al. ^102^ found that Ti particles can activate CHK2, trigger BRCA1 recruitment in oral epithelial cells, and activate DNA damage response (DDR) in epithelial cells. Some studies also propose that titanium and its oxides might bind to proteins, leading to hypersensitivity reactions such as itching, redness, and swelling of the skin. However, the connection between this and the development of inflammation necessitates further clarification.^103^ Autophagy is a conserved intracellular self-digestion system. In the large joint inflammation model, wear particles can cause abnormal cellular autophagic activity and autophagy dysfunction, promoting inflammation,^92,104–106^ indicating a potential role of autophagy in the development of peri-implantitis.

In summary, the influence of titanium particles on peri-implantitis seems to be multifaceted, involving a variety of interacting factors. This complexity should not be overlooked in future studies. Additionally, a substantial body of studies indicates that titanium particles play a very important role in the development of inflammation, making this an area ripe for further exploration.

## Possible solutions

**Fig. 4:**
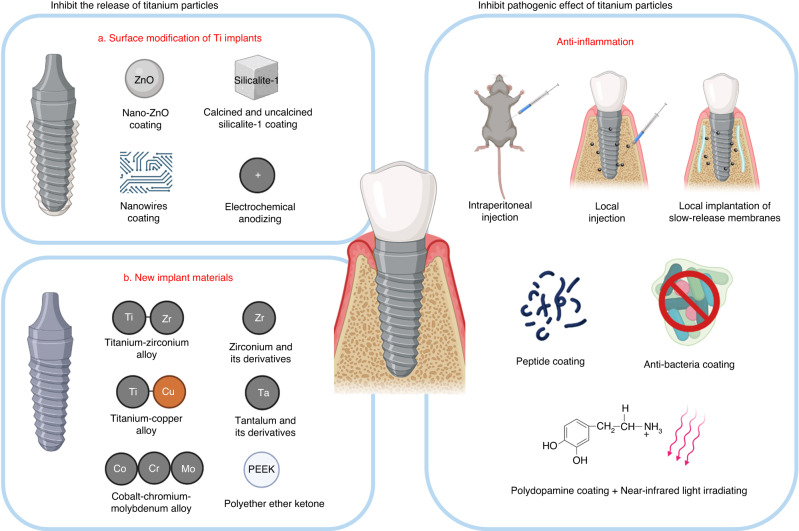
Possible solutions of the influence of titanium particles. Inhibit the release of titanium particles: the implant surface is modified by electrospinning, alkali thermal method and other technologies, so that the implant shows a coating film to inhibit the release of titanium particles; Use titanium zirconium, titanium copper and other alloy implants, improve implant materials, zirconium, tantalum, cobalt, chromium, molybdenum and other metal implants and PEEK composite polymer material implants; Inhibition of proinflammatory effect of titanium particles: by indicating modified loaded antibacterial and anti-inflammatory drugs, local injection, local placement of biofilm and other methods

The previous section aimed to summarize the role of titanium particles in the development of peri-implantitis, and provided an overview of current preventative methods and treatments. By understanding the pathogenic mechanism of titanium particles, we aim to propose potential methods, hoping to offer a new strategy for the prevention and treatment of peri-implantitis. Current strategies mainly focus on mitigating the release of titanium particles and inhibit their pathogenic effect. Many studies have achieved these goals through implant modification and the implementation of various factors. These methods may achieve both objectives simultaneously. In this review, to offer a more lucid perspective on potential treatment for titanium particle-induced peri-implantitis, we will discuss these two aspects separately, hoping to provide a clear direction on future treatment strategies (Fig. 4).

### Inhibit the release of titanium particles

At present, titanium implants inevitably release titanium particles into the surrounding tissues during implantation and long-term maintenance. To mitigate or slow the release of titanium particles, the first consideration is to surface-treat the implant to delay the corrosion rate and increase the resistance to friction.

A prevalent approach is to coat the implant surface, which considerably suppresses the release of titanium particles. Wang et al. ^107^ concluded that coating Nano-ZnO particles on implants promotes the corrosion resistance and antibacterial properties of implants. This surface modification strategy not only directly increase the corrosion resistance of implants, reducing the release of titanium particles, but it also indirectly affects the release of titanium particles as mentioned above. Wang et al. ^108^ found that applying calcined and uncalcined silicalite-1 coatings on titanium implants could bolster their corrosion resistance under inflammatory environment. In addition, alkali heat treatment and electrospinning can deposit nanowires on the titanium implant, forming nanoscale mesh structures that shield the underlying titanium from chemical corrosion.^109^ Refining the grain size to the nanometer level can enhance the mechanical and chemical stability of titanium implants by reducing the internal strain and establishing the titanium dioxide layer. Electrochemical anodizing (EA) is another method that can be employed to boost the corrosion resistance and bioactivity of titanium-based implants.^110^

Studies have also been conducted on the conversion of pure titanium implants into titanium alloy implants. Akimoto et al. ^111^ found that titanium-zirconium alloy implants, especially when the molar ratio of zirconium is less than 50%, exhibit good corrosion resistance which can reduce the release of titanium particles. Wu et al. ^112^ analyzed the implant created by blending copper and titanium using various hybrid methods. They assessed the implants’ potential antibacterial and corrosion -resistant capabilities and concluded that they have promising application prospects.

A more innovative strategy involves exploring new implant materials instead of relying on existing titanium-based implants. Wang et al. ^113^ concluded that tantalum and its derivatives show considerable application potential in orthopedic and dental implants due to their corrosion resistance, biocompatibility, bone integration ability and antibacterial properties. However, they noted that more research is required before these materials can be used in clinical application. Zirconium based implants have corrosion resistance and biocompatibility due to the formation of natural zirconia (ZrO_2_) film. Many studies have modified the surface of zirconium based implants using various physical, chemical and biological methods to make them more suitable for clinical application, which is a promising development direction.^114^ Implant materials made from cobalt-chromium-molybdenum alloy exhibit good mechanical properties and corrosion resistance, suggesting potential for future development in dentistry.^115^ Polyether ether ketone (PEEK) is a synthetic polymer material that has been used as a biomaterial in orthopedics for many years.^116^ Its similar mechanical and physical properties have given PEEK a wide range of application possibilities in oral implantology.^117^ However, PEEK implants can lead to poor fiber encapsulation and bone integration, improving the biological activity of polyether ether ketone dental implants without compromising their mechanical properties presents a major challenge and a promising research direction.^117,118^ Although exploring new materials can potentially avoid the release of titanium particles, it is crucial to ensure that these new material also meet other requirements for long-term maintenance of the implant. This makes the research more complex.

In conclusion, according to current understanding, inhibition of the release of titanium particles can be achieved by improving the metal alloy, or coating and modification of the coating to enhance the corrosion resistance and antibacterial properties of the implant. Although there is extensive research in this area, few have been successfully implemented in clinical practice, necessitating further exploration.

### Inhibit the pathogenic effect of titanium particles

Another direction to inhibit the development of titanium particle-induced peri-implantitis is to consider the pathogenic effect of titanium particles. Given that the specific role of titanium particles has not been fully clarified, the current treatment strategies primarily focus on the prevention and treatment of inflammation and osteolysis symptoms associated with peri-implantitis.

Eger et al. ^119^ demonstrated through experiments that either intraperitoneal injection or local implantation of slow-release membranes in the system around titanium implants, or local suppression of inflammation-related IL-1β, IL-6 and/or TNF-α expression could effectively inhibit the development of inflammation induced by titanium particles. Guo et al. ^120^ showed that a peptide segment adsorbed on titanium implants can increase the adhesion of osteoblasts and promote bone formation on the surface of titanium implants under inflammatory conditions. Jia et al. ^121^ also showed that applying an antibacterial coating to titanium implants could inhibit the absorption of bacteria and the formation of biofilm, and thus prevent inflammation. Similarly, Xiaoxiang et al. ^122^ showed that coating titanium implants with polydopamine nanoparticles, followed by a period of near-infrared light, can inhibit bacteria and reduce inflammation. Dextrorotatory-isoforms of amino acids (D-AAs) are vital components of the peptidoglycan in bacterial cell walls. They play a key role in bacterial adhesion to abiotic surfaces, promoting biofilm formation and its subsequent decomposition. This may be significant in preventing and treating peri-implantitis.^123^ Gulati et al. ^124^ have summarized methods for implant surface modification to promote bone formation and soft tissue integration. They present strategies including topographical, chemical, electrochemical, biological, and therapeutic modifications. Interestingly, they propose that electrochemically anodized titanium implants can influence the nano-morphology of the implant surface, enhance biological activity, and facilitate local therapeutic release. This could be an effective strategy for managing inflammation. In addition, considering the potential pathogenic signaling pathway of titanium particles, as summarized above, substances like melatonin,^86,93^ crocin,^94^ etc., may be used as potential therapeutic drugs, and more studies are needed to confirm this.

However, the existing strategy primarily involve incorporating antibacterial and anti-inflammatory substances into the implant to inhibit the development of peri-implantitis. Since these substances are mostly synthesized by the respective research groups, there is a lack of follow-up promotion and clinical application examples. Furthermore, the connection between this effect and titanium particles remains clear. The strategy of inhibiting the development of peri-implantitis by inhibiting the pathogenic effect of titanium particles presents a wide area for future research.

## Conclusion and future perspectives

Peri-implantitis is a prevalent biological complication that presents the most challenging treatment in oral implantology. The traditional symptomatic treatment method often fail to accurately address the cause, resulting in unsatisfactory outcomes. Therefore, it is crucial to identify the progression of peri-implantitis and discover ways to alleviate and obstruct the development of inflammation to optimize the prevention and treatment strategies. Throughout the whole implant treatment process, titanium implants continuously release titanium particles to surrounding tissues due to drill wear, friction between implant and bone surface, wear caused by biomechanical load, biological friction corrosion effect and other factors. The role of titanium particles in the progression of peri-implantitis may be underestimated. In recent years, some studies have shown that the concentration of titanium particles in the tissue at the peri-implantitis site is significantly increased. However, these studies lack continuity. Most only describe the phenomenon of increased titanium particle content, without further exploring the possible mechanism. Moreover, due to the absence of a unified standard, there is limited value of comparison between different researches, thus limiting the evidence for the further exploration of the mechanisms. Future studies urgently need to establish a standardized process of sampling and examination to provide a basis for the rule of titanium particle release at the peri-implantitis site and subsequent mechanism research. Simultaneously, by comprehending the patterns of titanium particle release around implants, future research can use the release and distribution of titanium particles as a reference to find ways to inhibit their release. This could be an effective approach to mitigating the impact of titanium particles.

In recent years, there have been many studies on the proinflammatory effects of titanium particles. Some suggest that an excess of these particles induces a foreign body reaction. However, the presence of titanium particles was also detected around healthy implants, indicating that the foreign body reaction alone could not explain the increased concentration of titanium particles at the peri-implantitis site. It’s worth noting that some epigenetic alterations may contribute to the pro-inflammatory effects of titanium particles, which vary depending upon the particle size. This requires a more precise detection method for the distribution of titanium particles around the implant, as described above. Drawing parallels with the adverse effects of wear particles after major joint replacement, titanium particles may regulate the development of inflammation and the process of osteolysis by affecting Wnt/β-catenin, NF-κB/RANKL/OPG and other signaling pathways. This offers promising directions for future research into the role of titanium particles in the peri-implantitis development. Studies also suggest a synergistic effect between titanium particles and bacteria in driving inflammation, hinting at a potential correlation between the influence of titanium particles and the risk factors for peri-implantitis. More longitudinal studies are needed to confirm this. In addition, Other areas of research such as autophagy, DNA damage, etc., further broaden our understanding of the role of titanium particles in the development of peri-implantitis. In future research, scholars should consider combining titanium particles with factors such as bacteria, autophagy, and DNA damage to explore potential synergistic effects. Signaling pathways related to inflammation development, such as Wnt/β-catenin and NF-κB/RANKL/OPG, require further attention to clarify the pathogenic mechanisms of titanium particles.

Because the role of titanium particles in the development of peri-implantitis remains to be clarified, means to inhibit their negative effects remain limited. Currently, the common method is to modify the surface of the implant. Techniques such as alkaline thermal method and electrostatic spinning technology are used to coat the implant, thereby inhibiting the release of titanium particles and potentially providing some anti-inflammatory effect. There is also ongoing research into innovative implant materials, including titanium alloys like titanium-zirconium and titanium-copper, which show potential for implant use. Other studies are considering tantalum, zirconium, peek biomaterials, etc. as alternatives to fundamentally mitigate the adverse effects of titanium particles. However, challenges abound in both implant modification and developing new implant materials. Factors such as biocompatibility of the implant, good bone bonding, adequate mechanical support, and the lack of other side effects of the new material all require further research. Moreover, titanium-based implants have proved to be commercially viable due to their excellent performance, even though the impact of titanium particles is not entirely established. This makes advancing research in this area particularly challenging. Another approach is to inhibit the pro-inflammatory effect of titanium particles by coating or loading the implant with anti-inflammatory and bacteriostatic drugs. However, this requires a deeper understanding of the role of titanium particles in the progression of peri-implantitis to achieve effective treatment. Therefore, a significant amount of work is still needed to better understand the role of titanium particles in both the area around the implant and the development of peri-implantitis. The mechanisms for both the inhibition of titanium particle release and the mitigation of their pathogenic effects remain largely unknown. These areas represent promising research directions that have yet to be fully explored. It is our hope that this review will provide new insights for further studies in this field.

### Supplementary information

Supplementary Information

## References

[1] Kotsakis GA, Olmedo DG (2021). Peri-implantitis is not periodontitis: Scientific discoveries shed light on microbiome-biomaterial interactions that may determine disease phenotype. Periodontol 2000.

[2] Jepsen S (2018). Periodontal manifestations of systemic diseases and developmental and acquired conditions: Consensus report of workgroup 3 of the 2017 World Workshop on the Classification of Periodontal and Peri-Implant Diseases and Conditions. J. Periodontol..

[3] Dixon DR, London RM (2019). Restorative design and associated risks for peri-implant diseases. Periodontol 2000.

[4] Komatsu K (2020). Discriminating microbial community structure between peri-implantitis and periodontitis with integrated metagenomic, metatranscriptomic, and network analysis. Front. Cell. Infect. Microbiol..

[5] Carcuac O (2013). Experimental periodontitis and peri-implantitis in dogs. Clin. Oral. Implants Res.

[6] Nakajima H, Okabe T (1996). Titanium in dentistry: development and research in the U.S.A. Dent. Mater. J..

[7] Sarraf M, Rezvani Ghomi E, Alipour S, Ramakrishna S, Liana Sukiman N (2022). A state-of-the-art review of the fabrication and characteristics of titanium and its alloys for biomedical applications. Bio-Des. Manuf..

[8] Franchi M (2004). Early detachment of titanium particles from various different surfaces of endosseous dental implants. Biomaterials.

[9] Schwarz F, Derks J, Monje A, Wang HL (2018). Peri-implantitis. J. Periodontol..

[10] Berglundh T (2018). Peri-implant diseases and conditions: Consensus report of workgroup 4 of the 2017 World Workshop on the Classification of Periodontal and Peri-Implant Diseases and Conditions. J. Clin. Periodontol..

[11] Delgado-Ruiz, R & Romanos, G. Potential causes of titanium particle and ion release in implant dentistry: A systematic review. *Int. J. Mol. Sci*. **19**, 10.3390/ijms19113585 (2018). 10.3390/ijms19113585 PMC6274707 30428596

[12] Romanos, GE, Fischer, GA & Delgado-Ruiz, R. Titanium wear of dental implants from placement, under loading and maintenance protocols. *Int. J. Mol. Sci*. **22**, 10.3390/ijms22031067 (2021). 10.3390/ijms22031067 PMC7865642 33494539

[13] Möhlhenrich SC, Modabber A, Steiner T, Mitchell DA, Hölzle F (2015). Heat generation and drill wear during dental implant site preparation: Systematic review. Br. J. Oral. Maxillofac. Surg..

[14] Rashad A (2013). Material attrition and bone micromorphology after conventional and ultrasonic implant site preparation. Clin. Oral. Implants Res.

[15] Alevizakos V, Mitov G, Ahrens AM, von See C (2021). The influence of implant site preparation and sterilization on the performance and wear of implant drills. Int. J. Oral. Maxillofac. Implants.

[16] Allsobrook OF, Leichter J, Holborrow D, Swain M (2011). Descriptive study of the longevity of dental implant surgery drills. Clin. Implant Dent. Relat. Res..

[17] Oliveira N, Alaejos-Algarra F, Mareque-Bueno J, Ferrés-Padró E, Hernández-Alfaro F (2012). Thermal changes and drill wear in bovine bone during implant site preparation. A comparative in vitro study: twisted stainless steel and ceramic drills. Clin. Oral. Implants Res.

[18] Möhlhenrich SC (2016). Influence of bone density and implant drill diameter on the resulting axial force and temperature development in implant burs and artificial bone: an in vitro study. Oral. Maxillofac. Surg..

[19] Barrak F (2022). Particle release from dental implants immediately after placement - An ex vivo comparison of different implant systems. Dent. Mater..

[20] Guan H, van Staden RC, Johnson NW, Loo YC (2011). Dynamic modelling and simulation of dental implant insertion process—A finite element study. Finite Elem. Anal. Des..

[21] Gao SS, Zhang YR, Zhu ZL, Yu HY (2012). Micromotions and combined damages at the dental implant/bone interface. Int. J. oral. Sci..

[22] Alqutaibi AY, Aboalrejal AN (2018). Microgap and micromotion at the implant abutment interface cause marginal bone loss around dental implant but more evidence is needed. J. Evid. Based Dent. Pract..

[23] Lopes PA (2018). Physicochemical and microscopic characterization of implant-abutment joints. Eur. J. Dent..

[24] Blum K (2015). Fatigue induced changes in conical implant-abutment connections. Dent. Mater..

[25] Gratton DG, Aquilino SA, Stanford CM (2001). Micromotion and dynamic fatigue properties of the dental implant-abutment interface. J. Prosthet. Dent..

[26] Binon PP (1996). The effect of implant/abutment hexagonal misfit on screw joint stability. Int. J. Prosthodont..

[27] Zipprich H, Weigl P, Ratka C, Lange B, Lauer HC (2018). The micromechanical behavior of implant-abutment connections under a dynamic load protocol. Clin. Implant Dent. Relat. Res..

[28] Huang, HH et al. Blood coagulation on titanium dioxide films with various crystal structures on titanium implant surfaces. *Cells* **11**, 10.3390/cells11172623 (2022). 10.3390/cells11172623 PMC9454428 36078030

[29] Addison O (2012). Do ‘passive’ medical titanium surfaces deteriorate in service in the absence of wear?. J. R. Soc., Interface.

[30] Valente ML, Lepri CP, dos Reis AC (2014). In vitro microstructural analysis of dental implants subjected to insertion torque and pullout test. Braz. Dent. J..

[31] Feng B (2002). Characterization of surface oxide films on titanium and bioactivity. J. Mater. Sci. Mater. Med..

[32] Alrabeah GO, Knowles JC, Petridis H (2016). The effect of platform switching on the levels of metal ion release from different implant-abutment couples. Int. J. oral. Sci..

[33] Ramel CF (2016). Surface roughness of dental implants and treatment time using six different implantoplasty procedures. Clin. Oral. Implants Res.

[34] Dhaliwal JS (2021). Microbial biofilm decontamination on dental implant surfaces: A mini review. Front. Cell. Infect. Microbiol..

[35] Louropoulou A, Slot DE, Van der Weijden FA (2012). Titanium surface alterations following the use of different mechanical instruments: a systematic review. Clin. Oral. Implants Res.

[36] Ronay V, Merlini A, Attin T, Schmidlin PR, Sahrmann P (2017). In vitro cleaning potential of three implant debridement methods. Simulation of the non-surgical approach. Clin. Oral. Implants Res.

[37] Mouhyi J, Dohan Ehrenfest DM, Albrektsson T (2012). The peri-implantitis: implant surfaces, microstructure, and physicochemical aspects. Clin. Implant Dent. Relat. Res.

[38] Mombelli A, Hashim D, Cionca N (2018). What is the impact of titanium particles and biocorrosion on implant survival and complications? A critical review. Clin. Oral. Implants Res.

[39] Wheelis SE (2016). Effects of decontamination solutions on the surface of titanium: investigation of surface morphology, composition, and roughness. Clin. Oral. Implants Res.

[40] Chen WQ, Zhang SM, Qiu J (2020). Surface analysis and corrosion behavior of pure titanium under fluoride exposure. J. Prosthet. Dent..

[41] Chen X (2020). Elucidating the corrosion-related degradation mechanisms of a Ti-6Al-4V dental implant. Dent. Mater..

[42] Barbieri M (2017). Corrosion behavior of dental implants immersed into human saliva: preliminary results of an in vitro study. Eur. Rev. Med. Pharmacol. Sci..

[43] Faverani LP (2014). Effect of bleaching agents and soft drink on titanium surface topography. J. Biomed. Mater. Res. B Appl. Biomater..

[44] Siddiqui DA (2019). Evaluation of oral microbial corrosion on the surface degradation of dental implant materials. J. Periodontol..

[45] Alhamad, M, Barão, VAR, Sukotjo, C, Cooper, LF & Mathew, MT. Ti-ions and/or particles in saliva potentially aggravate dental implant corrosion. *Materials (Basel, Switzerland)* **14**, 10.3390/ma14195733 (2021). 10.3390/ma14195733 PMC8510105 34640130

[46] Herbster M (2022). Microstructure-dependent crevice corrosion damage of implant materials CoCr28Mo6, TiAl6V4 and REX 734 under severe inflammatory conditions. J. Biomed. Mater. Res. B Appl. Biomater..

[47] Sridhar S (2015). In vitro investigation of the effect of oral bacteria in the surface oxidation of dental implants. Clin. Implant Dent. Relat. Res..

[48] Mathew MT (2012). What is the role of lipopolysaccharide on the tribocorrosive behavior of titanium?. J. Mech. Behav. Biomed. Mater..

[49] Safioti LM, Kotsakis GA, Pozhitkov AE, Chung WO, Daubert DM (2017). Increased Levels of Dissolved Titanium Are Associated With Peri-Implantitis - A Cross-Sectional Study. J. Periodontol..

[50] Suárez-López Del Amo F, Garaicoa-Pazmiño C, Fretwurst T, Castilho RM, Squarize CH (2018). Dental implants-associated release of titanium particles: A systematic review. Clin. Oral. Implants Res.

[51] Flatebø RS (2011). Mapping of titanium particles in peri-implant oral mucosa by laser ablation inductively coupled plasma mass spectrometry and high-resolution optical darkfield microscopy. J. Oral. Pathol. Med..

[52] He X (2016). Analysis of titanium and other metals in human jawbones with dental implants - A case series study. Dent. Mater..

[53] Weingart D (1994). Titanium deposition in regional lymph nodes after insertion of titanium screw implants in maxillofacial region. Int. J. Oral. Maxillofac. Surg..

[54] Wennerberg A, Jimbo R, Allard S, Skarnemark G, Andersson M (2011). In vivo stability of hydroxyapatite nanoparticles coated on titanium implant surfaces. Int. J. Oral. Maxillofac. Implants.

[55] Olmedo D, Guglielmotti MB, Cabrini RL (2002). An experimental study of the dissemination of Titanium and Zirconium in the body. J. Mater. Sci. Mater. Med..

[56] Guglielmotti MB (2015). Migration of titanium dioxide microparticles and nanoparticles through the body and deposition in the gingiva: an experimental study in rats. Eur. J. Oral. Sci..

[57] Fretwurst T (2016). Metal elements in tissue with dental peri-implantitis: a pilot study. Clin. Oral. Implants Res.

[58] Pettersson M, Pettersson J, Johansson A, Molin Thorén M (2019). Titanium release in peri-implantitis. J. Oral. Rehabil..

[59] Olmedo DG, Nalli G, Verdú S, Paparella ML, Cabrini RL (2013). Exfoliative cytology and titanium dental implants: a pilot study. J. Periodontol..

[60] Daubert DM, Pozhitkov AE, Safioti LM, Kotsakis GA (2019). Association of global DNA methylation to titanium and peri-implantitis: A case-control study. JDR Clin. Transl. Res..

[61] Rakic M (2022). Study on the immunopathological effect of titanium particles in peri-implantitis granulation tissue: A case-control study. Clin. Oral. Implants Res.

[62] Anderson JM, Rodriguez A, Chang DT (2008). Foreign body reaction to biomaterials. Semin. Immunol..

[63] Messous R (2021). Cytotoxic effects of submicron- and nano-scale titanium debris released from dental implants: an integrative review. Clin. Oral. Investig..

[64] Ivanovski S, Bartold PM, Huang YS (2022). The role of foreign body response in peri-implantitis: What is the evidence?. Periodontol 2000.

[65] Wang X, Li Y, Feng Y, Cheng H, Li D (2019). Macrophage polarization in aseptic bone resorption around dental implants induced by Ti particles in a murine model. J. Periodontal Res..

[66] Eger M, Sterer N, Liron T, Kohavi D, Gabet Y (2017). Scaling of titanium implants entrains inflammation-induced osteolysis. Sci. Rep..

[67] Ribeiro AR (2016). Trojan-like internalization of anatase titanium dioxide nanoparticles by human osteoblast cells. Sci. Rep..

[68] Lu X (2016). Short-term exposure to engineered nanomaterials affects cellular epigenome. Nanotoxicology.

[69] Malakootian M, Nasiri A, Osornio-Vargas AR, Faraji M (2021). Effect of titanium dioxide nanoparticles on DNA methylation of human peripheral blood mononuclear cells. Toxicol. Res..

[70] Ndika J (2019). Silver, titanium dioxide, and zinc oxide nanoparticles trigger miRNA/isomiR expression changes in THP-1 cells that are proportional to their health hazard potential. Nanotoxicology.

[71] Ichioka Y, Asa’ad F, Malekzadeh B, Westerlund A, Larsson L (2021). Epigenetic changes of osteoblasts in response to titanium surface characteristics. J. Biomed. Mater. Res. A.

[72] Freitag L (2023). Dental implant material related changes in molecular signatures in peri-implantitis - A systematic review and integrative analysis of omics in-vitro studies. Dent. Mater..

[73] Napetschnig J, Wu H (2013). Molecular basis of NF-κB signaling. Annu. Rev. Biophys..

[74] Bonnet N, Bourgoin L, Biver E, Douni E, Ferrari S (2019). RANKL inhibition improves muscle strength and insulin sensitivity and restores bone mass. J. Clin. Invest..

[75] Zhu S (2016). Strontium inhibits titanium particle-induced osteoclast activation and chronic inflammation via suppression of NF-κB pathway. Sci. Rep..

[76] Mishra V (2016). Titanium dioxide nanoparticles augment allergic airway inflammation and Socs3 expression via NF-κB pathway in murine model of asthma. Biomaterials.

[77] Yu X, Hong F, Zhang YQ (2016). Cardiac inflammation involving in PKCε or ERK1/2-activated NF-κB signalling pathway in mice following exposure to titanium dioxide nanoparticles. J. Hazard. Mater..

[78] Tang W (2020). Puerarin inhibits titanium particle-induced osteolysis and RANKL-induced osteoclastogenesis via suppression of the NF-κB signaling pathway. J. Cell. Mol. Med..

[79] Wachi T, Shuto T, Shinohara Y, Matono Y, Makihira S (2015). Release of titanium ions from an implant surface and their effect on cytokine production related to alveolar bone resorption. Toxicology.

[80] Krishnan V, Bryant HU, Macdougald OA (2006). Regulation of bone mass by Wnt signaling. J. Clin. Invest..

[81] Brandenburg J, Reiling N (2016). The Wnt blows: On the functional role of wnt signaling in mycobacterium tuberculosis infection and beyond. Front. Immunol..

[82] Schaale K, Neumann J, Schneider D, Ehlers S, Reiling N (2011). Wnt signaling in macrophages: augmenting and inhibiting mycobacteria-induced inflammatory responses. Eur. J. Cell Biol..

[83] Abaricia JO, Shah AH, Chaubal M, Hotchkiss KM, Olivares-Navarrete R (2020). Wnt signaling modulates macrophage polarization and is regulated by biomaterial surface properties. Biomaterials.

[84] Qu R (2019). Ghrelin fights against titanium particle-induced inflammatory osteolysis through activation of β-catenin signaling pathway. Inflammation.

[85] Wang, B *et al*. The effect of strontium ranelate on titanium particle-induced periprosthetic osteolysis regulated by WNT/β-catenin signaling in vivo and in vitro. *Biosci. Rep*. **41**, 10.1042/bsr20203003 (2021). 10.1042/BSR20203003 PMC7846966 33443286

[86] Ping Z (2017). Melatonin attenuates titanium particle-induced osteolysis via activation of Wnt/β-catenin signaling pathway. Acta Biomater..

[87] Zheng K (2021). Protective effects of sirtuin 3 on titanium particle-induced osteogenic inhibition by regulating the NLRP3 inflammasome via the GSK-3β/β-catenin signalling pathway. Bioact. Mater..

[88] Xiong L (2019). Acetyl-11-keto-β-boswellic acid attenuates titanium particle-induced osteogenic inhibition via activation of the GSK-3β/β-catenin signaling pathway. Theranostics.

[89] Ersahin T, Tuncbag N, Cetin-Atalay R (2015). The PI3K/AKT/mTOR interactive pathway. Mol. Biosyst..

[90] Zheng Z (2022). Involvement of PI3K/Akt signaling pathway in promoting osteogenesis on titanium implant surfaces modified with novel non-thermal atmospheric plasma. Front. Bioeng. Biotechnol..

[91] Wen Z (2022). MiR-92a/KLF4/p110δ regulates titanium particles-induced macrophages inflammation and osteolysis. Cell death Discov..

[92] Xian G (2020). Titanium particles induce apoptosis by promoting autophagy in macrophages via the PI3K/Akt signaling pathway. J. Biomed. Mater. Res. A.

[93] Wu Y (2021). Melatonin alleviates titanium nanoparticles induced osteolysis via activation of butyrate/GPR109A signaling pathway. J. nanobiotechnology.

[94] Zhu K (2019). Crocin inhibits titanium particle-induced inflammation and promotes osteogenesis by regulating macrophage polarization. Int. Immunopharmacol..

[95] Soler, MD et al. Titanium corrosion in peri-implantitis. *Materials (Basel, Switzerland)* **13**, 10.3390/ma13235488 (2020). 10.3390/ma13235488 PMC7730765 33276474

[96] Daubert D, Pozhitkov A, McLean J, Kotsakis G (2018). Titanium as a modifier of the peri-implant microbiome structure. Clin. Implant Dent. Relat. Res..

[97] Irshad M (2013). Influence of titanium on in vitro fibroblast-Porphyromonas gingivalis interaction in peri-implantitis. J. Clin. Periodontol..

[98] Weller J (2022). The role of bacterial corrosion on recolonization of titanium implant surfaces: An in vitro study. Clin. Implant Dent. Relat. Res..

[99] Yu TS (2004). Effect of titanium-ion on the growth of various bacterial species. J. Microbiol..

[100] Apaza-Bedoya K (2017). Synergistic interactions between corrosion and wear at titanium-based dental implant connections: A scoping review. J. Periodontal Res..

[101] Martin A, Zhou P, Singh BB, Kotsakis GA (2022). Transcriptome-wide gene expression analysis in peri-implantitis reveals candidate cellular pathways. JDR Clin. Transl. Res..

[102] Suárez-López Del Amo F (2017). Titanium activates the DNA damage response pathway in oral epithelial cells: A pilot study. Int. J. Oral. Maxillofac. Implants.

[103] Fage SW, Muris J, Jakobsen SS, Thyssen JP (2016). Titanium: a review on exposure, release, penetration, allergy, epidemiology, and clinical reactivity. Contact Dermat..

[104] Wang Z (2015). Autophagy mediated CoCrMo particle-induced peri-implant osteolysis by promoting osteoblast apoptosis. Autophagy.

[105] Chen W (2021). Autophagy inhibitors 3-MA and LY294002 repress osteoclastogenesis and titanium particle-stimulated osteolysis. Biomater. Sci..

[106] Camuzard O, Breuil V, Carle GF, Pierrefite-Carle V (2019). Autophagy involvement in aseptic loosening of arthroplasty components. J. Bone Jt. Surg. Am..

[107] Wang Z (2021). NanoZnO-modified titanium implants for enhanced anti-bacterial activity, osteogenesis and corrosion resistance. J. Nanobiotechnol..

[108] Wang J (2022). Enhanced corrosion resistance in an inflammatory environment and osteogenic properties of silicalite-1 coated titanium alloy implants. Colloids Surf. B, Biointerfaces.

[109] Zhu WQ (2019). Enhanced corrosion resistance of zinc-containing nanowires-modified titanium surface under exposure to oxidizing microenvironment. J. Nanobiotechnol..

[110] Guo, T, Scimeca, JC, Ivanovski, S, Verron, E & Gulati, K. Enhanced corrosion resistance and local therapy from nano-engineered titanium dental implants. *Pharmaceutics* **15**, 10.3390/pharmaceutics15020315 (2023). 10.3390/pharmaceutics15020315 PMC9963924 36839638

[111] Akimoto T (2018). Evaluation of corrosion resistance of implant-use Ti-Zr binary alloys with a range of compositions. J. Biomed. Mater. Res. B Appl. Biomater..

[112] Wu, Y et al. Recent advances in copper-doped titanium implants. *Materials (Basel, Switzerland)* **15**, 10.3390/ma15072342 (2022). 10.3390/ma15072342 PMC8999642 35407675

[113] Wang X, Ning B, Pei X (2021). Tantalum and its derivatives in orthopedic and dental implants: Osteogenesis and antibacterial properties. Colloids Surf. B, Biointerfaces.

[114] Chopra D, Jayasree A, Guo T, Gulati K, Ivanovski S (2022). Advancing dental implants: Bioactive and therapeutic modifications of zirconia. Bioact. Mater..

[115] Mace A, Khullar P, Bouknight C, Gilbert JL (2022). Corrosion properties of low carbon CoCrMo and additively manufactured CoCr alloys for dental applications. Dent. Mater..

[116] Kurtz SM, Devine JN (2007). PEEK biomaterials in trauma, orthopedic, and spinal implants. Biomaterials.

[117] Najeeb S, Zafar MS, Khurshid Z, Siddiqui F (2016). Applications of polyetheretherketone (PEEK) in oral implantology and prosthodontics. J. prosthodontic Res..

[118] Torstrick FB (2018). Porous PEEK improves the bone-implant interface compared to plasma-sprayed titanium coating on PEEK. Biomaterials.

[119] Eger M (2018). Mechanism and Prevention of Titanium Particle-Induced Inflammation and Osteolysis. Front. Immunol..

[120] Guo X (2022). Bioinspired peptide adhesion on Ti implants alleviates wear particle-induced inflammation and improves interfacial osteogenesis. J. Colloid Interface Sci..

[121] Wang J (2017). A decomposable silica-based antibacterial coating for percutaneous titanium implant. Int J. Nanomed..

[122] Ren X (2020). Eradicating infecting bacteria while maintaining tissue integration on photothermal nanoparticle-coated titanium surfaces. ACS Appl. Mater. interfaces.

[123] Caldwell M (2023). Promising applications of D-amino acids in periprosthetic joint infection. Bone Res..

[124] Gulati K (2023). Craniofacial therapy: advanced local therapies from nano-engineered titanium implants to treat craniofacial conditions. Int. J. oral. Sci..

[125] Wilson TG (2015). Foreign bodies associated with peri-implantitis human biopsies. J. Periodontol..

[126] Pettersson M (2017). Titanium ions form particles that activate and execute interleukin-1β release from lipopolysaccharide-primed macrophages. J. Periodontal Res..

[127] Nelson K (2020). Distribution and chemical speciation of exogenous micro- and nanoparticles in inflamed soft tissue adjacent to titanium and ceramic dental implants. Anal. Chem..

[128] Berryman Z (2020). Titanium particles: An emerging risk factor for peri-implant bone loss. Saudi Dent. J..

